# Rejected by an AI? Comparing job applicants’ fairness perceptions of artificial intelligence and humans in personnel selection

**DOI:** 10.3389/frai.2025.1671997

**Published:** 2025-11-04

**Authors:** Christine Malin, Jürgen Fleiß, Renate Ortlieb, Stefan Thalmann

**Affiliations:** ^1^Business Analytics and Data Science Center, University of Graz, Graz, Austria; ^2^Department of Human Resource Management, University of Graz, Graz, Austria

**Keywords:** artificial intelligence, fairness perceptions, job applicants, personnel selection, explanations

## Abstract

**Introduction:**

Artificial intelligence (AI) transforms personnel selection, but the application of AI raises fairness concerns and aversion towards AI. Although job applicants may perceive the selection process as fairer when they receive an explanation for the decision, scientific knowledge about AI-related fairness perceptions in this setting is limited. This paper investigates how job applicants perceive fairness of an AI-based personnel selection process considering explanations provided.

**Methods:**

The hypotheses are based on a theoretical framework about fairness and literature on algorithm aversion. Data were collected through a vignette-style method focusing on four personnel selection scenarios (*n* = 921).

**Results:**

We show that provided explanations increase job applicants’ perceptions of outcome fairness, process fairness, interpersonal treatment, and recommendation intention, irrespective of the decision being made by an AI or human.

**Discussion:**

We provide conclusions for algorithmic decision-making and discuss factors that need to be considered when adopting and designing AI so that AI is perceived as fair.

## 1 Introduction

Artificial intelligence (AI) is one of the most disruptive technologies, impacting business and society alike. However, the application of AI in human resource management (HRM) in general and in personnel selection in particular, is discussed controversially (Hunkenschroer and Luetge, 2022). Organizations are adopting AI in personnel selection to identify, screen, pre-select candidates, or interact with job applicants through conversational agents (Laurim et al., 2021). For example, automated CV screening, algorithmic aptitude tests, speech and facial expression analysis in video interviews, and chatbots are used. These approaches differ from traditional personnel selection techniques performed by humans, relying more on subjective assessments, personal interviews, and experiential knowledge. While AI-supported processes are based on data-driven models or are (partially) automated, thus promising efficiency and objectivity, personnel selection by humans is perceived as more personal, context-sensitive, and often fairer (Nabi, 2023). These differences are the central source of tension. Organizations are enthusiastic about the potential of AI-based personnel selection processes, while the perceptions of those affected by AI are less positive. Job applicants express aversion to AI in the process of personnel selection. Prior research has shown that job applicants tend to prefer human recruiters over AI and perceive them to be fairer (e.g., Tian et al., 2023). Therefore, it is important to systematically examine the tension between efficiency and fairness.

Experiences during the selection process shape the (future) employee-organization-relationship (Searle and Skinner, 2011) and job applicants’ trust in and their willingness to interact with AI-based work systems in their subsequent job position. Job applicants’ fairness perceptions can influence a variety of organizational factors, such as the perceived image of the organization or job applicants’ (re)application intentions (Gilliland, 1993). Thus, negative fairness perceptions of both hired and rejected job applicants can have negative consequences for organizations (Younis et al., 2024). Therefore, it is of great relevance to investigate job applicants’ fairness perceptions in AI-based personnel selection.

Literature on algorithm aversion provides explanations for the discrepancy between the high potential of AI and job applicants’ negative fairness perceptions, highlighting job applicants’ negative beliefs about AI (Jussupow et al., 2020). Job applicants were shown to be concerned that AI lacks intuition, making them perceive AI-based decisions as less fair than those made by human recruiters (Lee, 2018). One major reason behind these concerns is the black-box nature of AI (Castelvecchi, 2016), making it challenging even for experts to understand the decision logic of AI (Hunkenschroer and Luetge, 2022).

Research on explainable AI (XAI) addressed this challenge and offers tools to make the decision-making process, and the reasons behind AI decision comprehensible to users, thus enhancing their fairness perceptions in AI (Kim et al., 2023). However, to date little is known about the influence of explanations on fairness perceptions in the context of AI-based personnel selection. Gilliland et al. (2001) is one of the few studies that examined the influence of explanations on job applicants’ fairness perceptions in personnel selection and still often forms the starting point of research in this area. However, their research focused on personnel selection performed by humans. Considering the literature on algorithm aversion, stating that job applicants perceive AI and human recruiters differently regarding factors such as fairness (Jussupow et al., 2020), it is unclear to what extent the study’s results also apply to explanations of AI-based decisions. According to the XAI literature, AI-based explanations generally have the potential to positively influence fairness perceptions. However, we need to understand how AI-provided explanations affect fairness perceptions in the specific context of personnel selection. Hence, this study focuses on the following research question:

*“How do job applicants perceive the fairness of an AI-based personnel selection process considering explanations, compared to personnel selection performed by humans?”*

To address this question, we extend Gilliland et al. (2001) and investigate the effect of explanations by either AI or human recruiters on job applicants’ perceptions regarding various fairness constructs and their recommendation intentions. We draw on a vignette-style survey method combined with a two-factorial experimental design, for which we adapted Gilliland et al. (2001)‘s job application scenarios to an AI setting. By expanding Gilliland et al. (2001) to AI-based decision making, we contribute to the research on explanations on algorithmic decision-making, finding that AI-provided explanations can counteract job applicants’ resistance towards AI and thus support high-quality employee-organization-relationship. The provision of explanations is an essential feature for AI to be perceived as fair. We identify XAI factors that influence job applicants’ fairness perceptions of AI-based personnel selection.

## 2 Related work

### 2.1 Organizational justice theory

Fairness in the context of AI is discussed from a technical, legal and user perspective, with research on the latter often building on organizational justice theory. Organizational justice theory addresses how fair individuals perceive processes, outcomes, interactions, and justifications within an organization (Gilliland, 1993). In general, job applicants’ fairness perceptions have been shown to influence their behavioral intentions (McLarty and Whitman, 2016), both during and after personnel selection (Gilliland, 1993).

AI has been found to be perceived as comparatively unfair in personnel selection (e.g., Tian et al., 2023). Job applicants choosing between human and AI-based decisions have been shown to have lower trust in the technology and perceive it as less fair (Lee, 2018). Such a negative fairness perception of the hiring process arising from the use of AI could lead to negative perceptions of the company from potential future employees (Acikgoz et al., 2020) or job applicants dropping out of the selection process (Köchling et al., 2022). Thus, using AI in personnel selection can cause problems for personnel selection instead of enhancing it. It is thus important to understand under which conditions a negative perception of AI arises.

### 2.2 Algorithm aversion

Algorithm aversion captures individuals’ general preference for humans over AI-based decisions-makers (Berger et al., 2021; Mahmud et al., 2022), offering a theoretical basis explaining why job applicants trust human decisions more and perceive them as fairer over AI-based decisions (Burton et al., 2020), highlighting varying negative beliefs, intentions, and behaviors towards AI compared to human decision-makers (Daschner and Obermaier, 2022).

Reasons for the negative perception of AI in personnel selection include job applicants’ need for human involvement in a personnel selection process (Mirowska and Mesnet, 2022), a perceived lack of intuition and subjective judgment skills of AI (Lee, 2018), and the assumption that AI cannot recognize the job applicants’ uniqueness (Lavanchy et al., 2023). These concerns are closely related to the desire for transparency, which is also widely discussed in the IS community in relation to aversion to AI (Mahmud et al., 2022). Understanding how the decision was made by an AI could help alleviate negative beliefs about an AI’s capabilities and the resulting fairness perception, but AI’s black-box nature (Haque et al., 2023) results in often even experts not being able to fully understand the decision-making process (Hunkenschroer and Luetge, 2022).

Besides these challenges in perception, AI systems can also raise objective fairness concerns. Biased or incomplete training data can lead to certain groups of job applicants being systematically disadvantaged as historical selection patterns are reproduced (Rigotti and Fosch-Villaronga, 2024). The selection and weighting of evaluation criteria can also unintentionally favor certain demographics and reinforce structural inequalities. In addition, AI systems can make decisions that are difficult for humans to understand, thereby concealing discriminatory patterns. Even apparently neutral models can unintentionally reinforce existing inequalities among job applicants, for example through the way the models are optimized, which criteria they particularly weight, or how organizational requirements influence decision-making processes (Fernández-Martínez and Fernández, 2020). To better identify and understand such fairness concerns, special approaches have been developed to make the decision-making process of AI systems more transparent.

### 2.3 Recent studies on explainable AI

The technical research field of XAI developed tools for explaining AI-based decision-making (Kim et al., 2023). These explanations can be used to better understand the decision made by AI for the decision-maker (in our case the HR professional) and to communicate an explanation of the AI decision to others (in our case the job applicant) (Gashi et al., 2022). Such explanations are intended to positively affect users’ perceptions of fairness and trustworthiness of AI (Haque et al., 2023).

Although first empirical studies indicate that explanations can have a positive effect on job applicants’ fairness perception of AI-based personnel selection (Tian et al., 2023), it is questionable whether explanations can fully counteract algorithm aversion. Job applicants assign different attributes to AI and human-supported selection processes (Koch-Bayram and Kaibel, 2023), they perceive fairness based on different attributes (Lee and Baykal, 2017) and have different expectations about the performance of AI and humans (Burton et al., 2020).

Gilliland et al. (2001) is one of the few but also the best-known study that examined the influence of explanations on job applicants’ fairness perceptions. Their work builds on the fairness theory introduced by Folger and Cropanzano (1998) and investigates how different types of explanations in rejection letters affect job applicants’ fairness perceptions. The authors conducted two scenario-based studies and one field experiment in which respondents in the role of job applicants received a rejection letter after a negatively described application process (e.g., interview questions were too personal). The rejection letter included one out of three types of explanations for the rejection, and the participants answered questions based on this scenario about their fairness perceptions regarding the outcome of the personnel selection (outcome fairness), the application process (process fairness), the perceived interpersonal treatment, and their intention to recommend the organization. Overall, the findings revealed that both explanations justifying why the selected candidate was the most qualified and explanations justifying the hiring freeze with external conditions increased job applicants’ perceived fairness and recommendation intention.

Since Gilliland et al. (2001) conducted their study, personnel selection has transformed under the influence of technical advances (van Esch and Black, 2019), which has also changed job applicants’ fairness perceptions. However, Gilliland et al. (2001) has not lost its relevance for current research on fairness perception, as it remains the starting point for several recent studies investigating fairness perception in a technology-based personnel selection context (e.g., Tian et al., 2023). Thus, this paper aims at replicating and extending the fairness study of Gilliland et al. (2001) in the context of AI.

## 3 Hypotheses development

**Figure 1:**
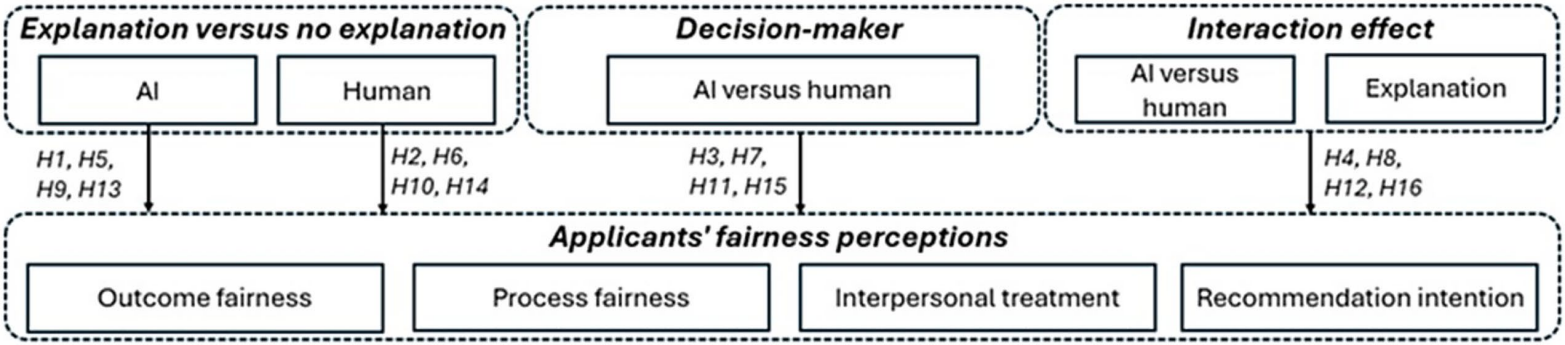
Research model.

We examine the effect of explanations of AI and human decisions on perceived fairness in the context of personnel selection (see Figure 1). Specifically, we replicate the hypotheses of Gilliland et al. (2001) on the influence of explanations on job applicants’ fairness perceptions of human-supported personnel selection. Building on algorithm aversion, we develop hypotheses on how job applicants perceive AI-based personnel selection and the effect of explanations. Since other studies (e.g., Fleiß et al., 2024) indicate that XAI explanations referring to objective criteria in comparison with other candidates are most effective, we will examine only the influence of this type of explanation on job applicants’ perception of fairness, adapting the explanations used by Fleiß et al. (2024) to the Gilliland et al. (2001) context of a rejection letter. Following organizational justice literature, we differentiate between distributive justice, i.e., how fair job applicants perceive the outcome of the selection process (outcome fairness), procedural justice, i.e., perceived fairness regarding the hiring process itself (process fairness), and interactional justice, i.e., the degree to which job applicants perceive that the hiring company has treated them respectfully during the hiring process (interpersonal treatment). Job applicants’ fairness perceptions influence their behavioral intentions such as their intention to recommend the hiring employer (i.e., recommendation intention) (Gilliland, 1993).

### 3.1 Outcome fairness

Gilliland et al. (2001) assumed that rejection letters that barely explain the selection decision can lead to negative reactions from job applicants, often in the form of anger towards the hiring company, resulting in lower perceived outcome fairness. Studies in justice research indicate that job applicants react more positively to negative decisions on their applications when explanations are provided. The authors showed this effect, especially for rejection letters that involve an explanation including why the person who received the job is more qualified than the rejected job applicants. Thus, the rejected job applicants are less likely to imagine an alternative setting in which they would have been hired.

As Gilliland et al. (2001) argue that explanations for a rejection decision made by a human recruiter increase job applicants’ perception of outcome fairness, we investigate whether this effect is also valid for rejection decisions made by an AI. XAI research indicates a positive impact of AI-provided explanations on the fairness perception. As algorithm aversion could counteract this positive effect of explanations in the AI scenario, AI-based explanations are expected to have a smaller impact on perceived outcome fairness than explanations provided by a human recruiter. Those assumptions are in line with results from previous studies. Lavanchy et al. (2023) highlighted in scenario experiments that job applicants may have concerns about outcome fairness when using AI in personnel selection, as they fear that AI is unable to identify job applicants’ unique skills. Job applicants’ negative fairness perceptions of the AI-based selection process persisted regardless of whether the hiring outcome is a rejection or an acceptance. In contrast, Mirowska and Mesnet (2022) showed AI use can also be beneficial for outcome fairness. When job applicants received a rejection, they perceived the AI-based decision as fair because they assumed that AI evaluates more objectively compared to human recruiters, considers mainly job-related and relevant characteristics for the evaluation, and is accurate. However, only a few studies investigated to what extent job applicants’ perceptions of outcome fairness of an AI-based decision differ in direct comparison to a decision made by a human recruiter (e.g., Narayanan et al., 2023; Tian et al., 2023). Limited research and partly contradictory findings make it difficult to observe comparative patterns. Given this background, we adapt the hypotheses of Gilliland et al. (2001) for our study as follows:

*H1*: Job applicants who interacted with an AI and received a rejection letter with an explanation will perceive greater outcome fairness than those who received a rejection letter without an explanation.

*H2*: Job applicants who interacted with a human recruiter and received a rejection letter with an explanation will perceive greater outcome fairness than those who received a rejection letter without an explanation.

*H3*: Job applicants who interacted with a human recruiter will perceive greater outcome fairness than those who interacted with an AI.

*H4*: A rejection letter with an explanation has a stronger effect on the perceived outcome fairness of job applicants who interacted with a human recruiter than those who interacted with an AI.

### 3.2 Process fairness

Gilliland et al. (2001) assumed that job applicants’ negative experiences during the personnel selection process lead them to perceive the process as unfair, while explanations in principle have the potential to increase fairness perceptions. For this effect to occur, the authors presupposed that the provided explanations had to address the personnel selection process. Since explanations expressing why the selected job applicant was the most qualified person for the open position do not meet this requirement, they expected no effect on the fairness construct.

Contrary to expectations that explanations, even if they did not directly address the selection process, positively influenced the perception of process fairness. We thus investigate the impact of explanations on job applicants’ process fairness perceptions in AI-based personnel selection. As XAI research indicates that explanations increase fairness perception in general, we hypothesize to observe the positive effect of the explanations identified by Gilliland et al. (2001) also in an AI setting. Czernietzki et al. (2023) found this positive influence of sharing information on job applicants’ perceptions of process fairness. However, job applicants’ perceptions of process fairness of AI-based personnel selection are rather mixed (Narayanan et al., 2023), making it unclear how explanations from AI influence job applicants’ perception of this fairness construct. While some studies (Öttning and Maier, 2018; Suen et al., 2019) observed no differences in job applicants’ perceptions of process fairness when confronted with an AI instead of a human in personnel selection, others (Mirowska and Mesnet, 2022; Kleiner et al., 2023; Cai et al., 2024) showed that job applicants perceived an AI-based evaluation of interviews or resume screening as more procedurally fair than those performed by a human recruiter. Several factors of algorithm aversion may lead to more negative perceptions of process fairness among AI compared to human recruiters. Job applicants may assume that AI does not have the necessary skills to assess personal qualities (e.g., charisma) (Mirowska and Mesnet, 2022) and social competencies (Acikgoz et al., 2020), reducing them to quantifiable performance data (Newman et al., 2020). This and the concern of being limited in their ability to express themselves to AI, can foster the concern that AI will negatively affect their chance for performing (Acikgoz et al., 2020). Job applicants perceive less fairness for tasks that they associate with the need for human skills, such as subjective judgment, rather than mechanical skills, such as processing quantitative data for objective assessments (Narayanan et al., 2023; Lee, 2018). Since our study examines the use of AI for personnel selection, AI is used for tasks that are typically considered complex and require human skills. Hence, we expect that job applicants will perceive the use of AI as less procedurally fair than when human recruiters are involved. Studies such as Tian et al. (2023) indicate that perceived process fairness of a decision is greater for an AI with explanation compared to a human decision without explanation. Thus, we assume that explanations generally have a positive impact on the perception of process fairness, but at the same time, that due to algorithm aversion counteracting the positive effect of explanations in the AI scenario only, AI-based explanations have a smaller impact on perceived process fairness than explanations provided by a human recruiter. This leads us to the following (adopted) hypotheses:

*H5*: Job applicants who interacted with an AI and received a rejection letter with an explanation will perceive greater process fairness than those who received a rejection letter without an explanation.

*H6*: Job applicants who interacted with a human recruiter and received a rejection letter with an explanation will perceive greater process fairness than those who received a rejection letter without an explanation.

*H7*: Job applicants who interacted with a human recruiter will perceive greater process fairness than those who interacted with an AI.

*H8*: A rejection letter with an explanation has a stronger effect on the perceived process fairness of job applicants who interacted with a human recruiter than those who interacted with an AI.

### 3.3 Interpersonal treatment

Gilliland et al. (2001) examined the effect of explanations on job applicants’ perceptions of interpersonal treatment. They hypothesized that explanations conveyed an honest and open approach of the company, resulting in higher levels of positive interpersonal treatment compared to rejection letters without explanations.

We investigate whether this positive effect of explanations on perceived interpersonal treatment also holds for AI. We assume that interpersonal treatment is perceived more negatively for AI compared to human recruiters due to algorithm aversion (Nørskov et al., 2020; Kaibel et al., 2019). Noble et al. (2022) showed that interpersonal treatment is perceived as worse in AI-based application and resume screening; Acikgoz et al. (2020) indicated the same negative effect in the context of AI-based interviews. This can make job applicants feel that the company does not care about its employees (Acikgoz et al., 2020), similar to the negative effect of a lack of direct communication (Noble et al., 2022). Also, job applicants assume that they can no longer adapt their self-presentation tactics to the reactions of the interviewer when using AI as they can with human decision-makers (Nørskov et al., 2020). Hence, we adapt the hypotheses from Gilliland et al. (2001) as follows:

*H9*: Job applicants who interacted with an AI and received a rejection letter with an explanation will perceive better interpersonal treatment than those who received a rejection letter without an explanation.

*H10*: Job applicants who interacted with a human recruiter and received a rejection letter with an explanation will perceive better interpersonal treatment than those who received a rejection letter without an explanation.

*H11*: Job applicants who interacted with a human recruiter will perceive better interpersonal treatment than those who interacted with an AI.

*H12*: A rejection letter with an explanation has a stronger effect on the perceived interpersonal treatment of job applicants who interacted with a human recruiter than those who interacted with an AI.

### 3.4 Recommendation intentions

Job applicants’ fairness perceptions have an impact on their intention to recommend jobs to others (McLarty and Whitman, 2016). Therefore, Gilliland et al. (2001) examined alongside the influence of explanations on individual fairness constructs also their influence on job applicants’ recommendation intentions. Although the authors did not formulate a specific hypothesis to investigate this relationship, the study showed that explanations justifying why the selected job applicant was the most qualified increased job applicants’ recommendation intention.

Previous research on job applicants’ fairness perceptions in AI-assisted personnel selection focused on examining the individual fairness constructs, neglecting behavioral intentions. One of the few studies doing so is Nørskov et al. (2020), which found that job applicants were more likely to intend to recommend the company to other job seekers when the interview was conducted by a human recruiter rather than by an AI. Algorithm aversion drives negative fairness perception, leading to decreased recommendation intention. Therefore, we expect AI-based explanations to have a smaller impact on perceived recommendation intentions compared to those provided by human recruiters:

*H13*: Job applicants who interacted with an AI and received a rejection letter with an explanation will have higher recommendation intention than those who received a rejection letter without an explanation.

*H14*: Job applicants who interacted with a human recruiter and received a rejection letter that included an explanation will have higher recommendation intention than those who received a rejection letter without an explanation.

*H15*: Job applicants who interacted with a human recruiter will have higher recommendation intention than those who interacted with an AI.

*H16*: A rejection letter with an explanation has a stronger effect on the perceived recommendation intention of job applicants who interacted with a human recruiter than those who interacted with an AI.

## 4 Procedure

We conducted a survey experiment comprising four vignettes, varying the two independent variables—whether an explanation for the rejection of the application was provided or not, and AI-based personnel selection versus human recruiter.

A quota-representative sample (*n* = 1,312) of the Austrian working-age population, concerning gender, age, province of residence, and educational attainment, was recruited through an ISO 20252:2019 certified online panel from Nortstat.

We excluded 390 respondents who exceeded the actual average Austrian retirement age of 59 years and one respondent due to incomplete responses, resulting in a final sample of *n* = 921 respondents with an average age of 39.48 years and 55.2% were women. Highest educational attainments ranged from compulsory school to university degree (18.7% compulsory school, 34.3% apprenticeship, 12.8% vocational secondary school, 16.2% high school, and 18.0% university or higher education degree).

Respondents were randomly assigned to one of four scenarios, in which they, in the role of a job applicant, interacted with either an AI or a human recruiter and received a rejection letter that included either no explanation or one explaining why the hired candidate was more qualified. Thus, each respondent received one of four scenario descriptions: (1) AI without explanation, (2) AI with explanation, (3) Human without explanation, and (4) Human with explanation.

The description of the scenarios and the rejection letters matched the original version of Gilliland et al. (2001), which we adapted to the local context of the study and translated into German. In all scenarios, we asked the respondents to imagine themselves in the role of Ms./Mr. Huber, who applied for a position as senior marketing manager. Their experience throughout the job application process was described as less than positive (e.g., job interview questions perceived as too personal). Subsequently, respondents were told to imagine receiving a rejection email.

After this introduction, half of all the respondents were instructed that they should imagine that the job interview and the selection of the most suitable job applicants for the second round of job interviews was conducted by an AI-based conversational agent (AI scenarios) and the other half that it was done by a human recruiter (human recruiter scenarios). We choose conversational agents to provide an AI context in our study, as they are one of the best-known AI applications that interact directly with job applicants during the personnel selection process (Diederich et al., 2020). We provided the respondents with a definition of an AI-based conversational agent and an animated example as part of the AI scenarios to ensure that all respondents have a common knowledge.

Half of the respondents in each AI and human recruiter scenario were informed that they received a rejection letter without an explanation, and the other half were informed that they received one that included one justifying the selection of the top candidates with their at least 15 years of domain-specific work experience as well as having senior management and industry-related experience. Then, all respondents completed a questionnaire. The questionnaire corresponded to the original version of Gilliland et al. (2001), which we adapted to the local context of our study, and to an AI context.

All dependent variables use a five-point rating from (1) “strongly disagree” to (5) “fully agree.” Outcome fairness measured how fair job applicants perceived the personnel selection decision (four items, e.g., “Given the situation, I feel the company made the right personnel selection decision”; Cronbach’s *α* = 0.91). Process fairness measured how fair job applicants perceived the personnel selection process (four items, e.g., “Whether or not I got the job, I feel the selection process was fair”; Cronbach’s *α* = 0.86). Interpersonal treatment measured how job applicants perceived the way they were treated during the application process (four items, e.g., “I was treated with a high degree of respect and sincerity”; Cronbach’s *α* = 0.85). Recommendation intention measured how likely they would recommend the company to others (three items, e.g., “I would recommend the company to my friends”; Cronbach’s *α* = 0.87). Our control variables are gender (1 = man), age (years), educational attainment (1 = compulsory school, 2 = apprenticeship, 3 = vocational secondary school, 4 = high school, and 5 = university or higher education degree), and perceived discrimination. The latter was measured by counting in how many of six different situations participants state that they were discriminated in the past (e.g., “Have you ever been fired from a job for unfair reasons?”).

## 5 Results

### 5.1 Descriptive results

Table 1 presents means and standard deviations, and the results of analyses of variance (ANOVAs) for the four dependent variables by scenario. Overall, the ANOVAs revealed statistically significant differences between the scenarios. For all four dependent variables, mean values are lowest for AI without explanation, ranging from 1.49 for *recommendation intention* to 1.86 for *outcome fairness*. *Outcome fairness*, *process fairness*, *interpersonal treatment*, and *recommendation intention* are consistently rated lowest when interacting with an AI and receiving a rejection letter without an explanation. Contrariwise, mean values are consistently highest for all dependent variables in Human with explanation. Here, mean values range from 1.94 for *recommendation intention* to 2.32 for *process fairness*.

**Table 1:** Descriptive statistics.

	AI without explanation	AI with explanation	Human without explanation	Human with explanation	Analysis of variance (ANOVA)
Outcome fairness	1.86 (0.70)	2.18 (0.71)	2.18 (0.64)	2.23 (0.56)	*F* (3, 917) = 15.14, *p* < 0.001
Process fairness	1.70 (1.10)	2.10 (1.09)	2.12 (0.91)	2.32 (0.87)	*F* (3, 917) = 15.22, *p* < 0.001
Interpersonal treatment	1.69 (1.01)	2.21 (0.97)	2.06 (0.94)	2.26 (0.85)	*F* (3, 917) = 17.11, *p* < 0.001
Recommendation intention	1.49 (1.09)	1.86 (1.18)	1.75 (1.07)	1.94 (1.03)	*F* (3, 917) = 7.48, *p* < 0.001

The table displays means and standard deviations (in parentheses), and ANOVA results for the dependent variables in the respective scenarios. The dependent variables range from 1 to 5, with higher values indicating higher perceived fairness and recommendation intention. The number of observations is *n* = 921.

While respondents consistently rated all dependent variables worst for AI without explanation and best for Human with explanation, evaluations for of the dependent variables for AI with explanation and Human without explanation show a mixed pattern. For both *outcome fairness* and *process fairness*, mean evaluations are similar for AI with an explanation (M = 2.18 & M = 2.10, respectively) and by a human recruiter without an explanation (M = 2.18 & M = 2.12, respectively). Evaluations of *interpersonal treatment* and *recommendation intention* were rated even higher for an AI with an explanation (M = 2.21 & M = 1.86, respectively) compared to a human recruiter without an explanation (M = 2.06 & M = 1.75, respectively). Explanations can thus increase positive evaluations of the interaction with, and decisions made by AI to the level of decisions made by a human recruiter without giving an explanation. In the case of *interpersonal treatment* and *recommendation intentions* the evaluations of decisions made by AI increase above those of decisions made by a human recruiter without explanation and even come close to the scenario with the highest rating, human with explanation.

Finally, in general, explanations have a positive effect on job applicants’ fairness perceptions, perceived *interpersonal treatment*, and *recommendation intention*, irrespective of the interaction with and decision being made by an AI or a human recruiter. When an explanation was given, mean ratings increase for all dependent variables, with the increase ranging from 0.32 to 0.52 for decisions made by an AI and from constant to 0.2 for decisions made by a human recruiter.

### 5.2 Hypotheses testing

We conducted OLS regression analyses to test our hypotheses that, in both AI-based personnel selection processes and those conducted by human recruiters, providing an explanation for the rejection leads to greater *outcome fairness* (H1 & H2), greater *process fairness* (H5 & H6), better *interpersonal treatment* (H9 & H10), and higher *recommendation intention* (H13 & H14). Table 2 presents the results (Models 1 to 4).

**Table 2:** Results of regression analysis.

	All scenarios				Scenarios involving an AI				Scenarios involving a human recruiter			
	Outcomefairness	Processfairness	Inter-personaltreatment	Recom-mendationintention	Outcomefairness	Processfairness	Inter-personaltreatment	Recom-mendationintention	Outcomefairness	Processfairness	Inter-personaltreatment	Recom-mendationintention
	(Model 1)	(Model 2)	(Model 3)	(Model 4)	(Model 1a)	(Model 2a)	(Model 3a)	(Model 4a)	(Model 1b)	(Model 2b)	(Model 3b)	(Model 4b)
AI with explanation	0.323^***^ (0.060)	0.394^***^ (0.091)	0.526^***^ (0.087)	0.372^***^ (0.100)	0.336^***^ (0.065)	0.404^***^ (0.101)	0.546^***^ (0.092)	0.383^***^ (0.105)				
Human without explanation	0.323^***^ (0.061)	0.416^***^ (0.092)	0.372^***^ (0.087)	0.262^***^ (0.100)								
Human with explanation	0.371^***^ (0.061)	0.614^***^ (0.092)	0.561^***^ (0.087)	0.437^***^ (0.100)					0.051 (0.055)	0.195^**^ (0.081)	0.185^**^ (0.082)	0.169^*^ (0.095)
Education (Ref. = compulsory school)												
Apprenticeship	−0.160^**^ (0.063)	−0.243^**^ (0.095)	−0.203^**^ (0.090)	−0.215^**^ (0.104)	−0.048 (0.095)	−0.177 (0.148)	−0.068 (0.135)	−0.042 (0.154)	−0.284^***^ (0.082)	−0.313^***^ (0.120)	−0.334^***^ (0.121)	−0.387^***^ (0.140)
Vocational secondary school	−0.006 (0.079)	−0.157 (0.120)	−0.087 (0.114)	−0.199 (0.131)	0.163 (0.119)	−0.034 (0.185)	0.173 (0.168)	−0.032 (0.192)	−0.182^*^ (0.104)	−0.287^*^ (0.154)	−0.378^**^ (0.154)	−0.384^**^ (0.179)
High school	−0.199^***^ (0.073)	−0.398^***^ (0.110)	−0.335^***^ (0.104)	−0.369^***^ (0.120)	−0.185^*^ (0.112)	−0.315^*^ (0.173)	−0.204 (0.158)	−0.208 (0.180)	−0.219^**^ (0.093)	−0.466^***^ (0.137)	−0.450^***^ (0.137)	−0.525^***^ (0.160)
University degree	−0.179^**^ (0.071)	−0.343^***^ (0.107)	−0.369^***^ (0.102)	−0.399^***^ (0.117)	−0.097 (0.110)	−0.300^*^ (0.171)	−0.234 (0.156)	−0.240 (0.178)	−0.258^***^ (0.090)	−0.385^***^ (0.132)	−0.501^***^ (0.132)	−0.559^***^ (0.154)
Age	−0.005^***^ (0.002)	−0.009^***^ (0.003)	−0.004 (0.003)	−0.006^**^ (0.003)	−0.007^**^ (0.003)	−0.015^***^ (0.004)	−0.009^**^ (0.004)	−0.010^**^ (0.005)	−0.003 (0.002)	−0.004 (0.004)	0.001 (0.004)	−0.003 (0.004)
Gender (woman)	−0.086^**^ (0.043)	−0.228^***^ (0.066)	−0.192^***^ (0.062)	−0.222^***^ (0.072)	−0.133^**^ (0.067)	−0.235^**^ (0.104)	−0.219^**^ (0.094)	−0.273^**^ (0.108)	−0.038 (0.056)	−0.224^***^ (0.082)	−0.158^*^ (0.082)	−0.165^*^ (0.095)
Experienced discrimination	0.030^**^ (0.014)	0.037^*^ (0.021)	0.048^**^ (0.020)	0.090^***^ (0.023)	0.049^**^ (0.022)	0.042 (0.035)	0.031 (0.032)	0.075^**^ (0.036)	0.016 (0.018)	0.039 (0.026)	0.072^***^ (0.026)	0.105^***^ (0.031)
Constant	2.184^***^ (0.101)	2.372^***^ (0.154)	2.076^***^ (0.146)	1.962^***^ (0.167)	2.170^***^ (0.149)	2.515^***^ (0.231)	2.158^***^ (0.210)	2.010^***^ (0.240)	2.507^***^ (0.126)	2.613^***^ (0.185)	2.313^***^ (0.186)	2.159^***^ (0.216)
Observations	921	921	921	921	462	462	462	462	459	459	459	459
*R* ^2^	0.082	0.094	0.092	0.076	0.095	0.079	0.099	0.067	0.046	0.072	0.080	0.087
Adjusted *R*^2^	0.072	0.084	0.083	0.066	0.079	0.063	0.084	0.051	0.029	0.056	0.063	0.070
Residual Std. error	0.645 (df = 910)	0.978 (df = 910)	0.929 (df = 910)	1.066 (df = 910)	0.693 (df = 453)	1.077 (df = 453)	0.981 (df = 453)	1.119 (df = 453)	0.591 (df = 450)	0.871 (df = 450)	0.871 (df = 450)	1.013 (df = 450)
*F* statistic	8.138^***^ (df = 10; 910)	9.431^***^ (df = 10; 910)	9.275^***^ (df = 10; 910)	7.521^***^ (df = 10; 910)	5.952^***^ (df = 8; 453)	4.870^***^ (df = 8; 453)	6.251^***^ (df = 8; 453)	4.093^***^ (df = 8; 453)	2.722^***^ (df = 8; 450)	4.389^***^ (df = 8; 450)	4.875^***^ (df = 8; 450)	5.329^***^ (df = 8; 450)

^*p, **p, ***p < 0.01.^ The table displays the unstandardized coefficients of OLS regression models with dummy variables for the different scenarios as independent variables (reference category is AI without explanation). Standard errors are given in parentheses. Models 1 to 4 are based on the pooled sample of all four scenarios, while Models 1a to 4a are based on the two pooled AI scenarios or Models 1b to 4b on the two pooled human scenarios.

For all four dependent variables, the coefficients are statistically significant for the scenarios AI with explanation, Human without explanation, and Human with explanation, with AI without explanation serving as the baseline scenario. This indicates, together with the positive values of the regression coefficients, that our respondents in both scenarios with explanation and the scenario with a human recruiter without explanation rate *outcome fairness*, *process fairness*, *interpersonal treatment*, and *recommendation intention* higher as compared to the scenario AI without explanation (all *p* < 0.001). This result resembles the previously established pattern, based on the descriptive results, of lowest evaluations in all dependent variables for the scenario of an AI without an explanation. The same holds true for the Human with explanation scenario showing the highest ratings in all dependent variables. Furthermore, when an explanation is provided in an AI-based personnel selection process, job applicants perceive greater *outcome fairness* (*b* = 0.336, *p* < 0.001), *process fairness* (*b* = 0.404, *p* < 0.001), *interpersonal treatment* (*b* = 0.546, *p* < 0.001), as well as higher *recommendation intention* (*b* = 0.383, *p* < 0.001) compared to when no explanation is provided. Scenarios involving a human recruiter yield similar results. However, job applicants did not perceive significantly higher *outcome fairness* (*b* = 0.051, *p* = 0.360) when a human recruiter provided an explanation for the job rejection decision, compared to without explanation. Thus, our results support H1, H5, H6, H9, H10, H13, and H14, but not H2.

Table 2 also provides results concerning our hypotheses that, when the personnel selection process is conducted by a human recruiter instead of an AI, job applicants perceive greater *outcome fairness* (H3), greater *process fairness* (H7), better *interpersonal treatment* (H11), and higher *recommendation intention* (H15). When an explanation was provided, larger regression coefficients for decisions made by an AI indicate that these are evaluated higher regarding *outcome fairness* (*b* = 0.323, *p* < 0.001 compared to *b* = 0.323, *p* = <0.001), for *interpersonal treatment* (*b* = 0.526, *p* = <0.001 compared to *b* = 0.372, *p* = <0.001), and for *recommendation intention* (*b* = 0.372, *p* < 0.001 compared to *b* = 0.262, *p* = 0.010) than decisions made by a human recruiter without an explanation. Only *process fairness* (*b* = 0.394, *p* < 0.001) was perceived lower in the scenario of an AI with an explanation as compared to the scenario Human without explanation. Coefficients indicate a significant positive impact of explanations on evaluations of decisions made by an AI, increasing them up to and, in some instances, even above the level of decisions made by a human recruiter without explanations. Thus, our findings support H3, H7, H11, and H15 only in certain cases: when human recruiters provide an explanation for the rejection decision, all hypotheses regarding greater *outcome fairness* (H3), greater *process fairness* (H7), better *interpersonal treatment* (H11), a higher *recommendation intention* (H15), when the personnel selection is conducted by a human recruiter compared to an AI are supported. The support for all Hypotheses also holds in all cases where no explanation is provided in an AI-based personnel selection process. However, when an explanation for the AI-based decision is provided, job applicants perceive an AI-based personnel selection process regarding *outcome fairness* as equally high or *interpersonal treatment* and *recommendation intention* as higher compared to a human recruiter-supported personnel selection process without explanation, thus rejecting H3, H11, and H15, and supporting H7.

To investigate whether an explanation for rejection has a stronger effect on the perceived *outcome fairness* (H4), *process fairness* (H8), *interpersonal treatment* (H12), and *recommendation intention* (H16) of job applicants who interacted with a human recruiter compared to an AI, for each of the four corresponding dependent variables we estimate OLS regressions containing a dummy variable for decision-maker (AI versus human) an for whether an explanation was given as well as their interaction term. We find a significant negative effect for the interaction between explanation and decision-maker for the regression on *outcome fairness* (*b* = −0.264, *p* = 0.002) and *interpersonal treatment* (*b* = −0.324, *p* = 0.009). Thus, an explanation has a significantly stronger effect on job applicants’ perceived *outcome fairness* and *interpersonal treatment*, when the personnel selection process is conducted by an AI as compared to a human recruiter. We find no significant interaction effects of explanation and decision-maker for *process fairness* (*b* = −0.194, *p* = 0.142) and *recommendation intention* (*b* = −0.193, *p* = 0.181).

Consequently, our results reject H4 and H12, but support H8 and H16. Note that, however, the direction of all interaction effects is consistently negative, suggesting that the interaction effect may be present in all treatments but varying in strength.

Supplementary to testing our hypotheses, as the regression analyses revealed the influences of some of the control variables on job applicants’ fairness perceptions, perceived *interpersonal treatment*, and *recommendation intention*, we examined sociodemographic characteristics in more detail. To do so, we ran separate regressions for the two AI scenarios vis-a-vis the two human recruiter scenarios. Models 1a to 4a in Table 2 show the results for the two pooled AI scenarios, while models 1b to 4b in Table 2 show those of the two pooled human scenarios.

For three of the pooled models (Model 1, 2, and 4), we find statistically significant negative coefficients for age. With increasing age, job applicants’ perceptions of *outcome fairness* (*b* = −0.005, *p* = 0.006), *process fairness* (*b* = −0.009, *p* = 0.001), and *recommendation intention* (*b* = −0.006, *p* = 0.036) decrease. However, the negative age effects disappear in the models based on only observations where the interaction and decision were made by a human recruiter, while they become stronger in the regressions based on observations where the decision were made by an AI and also become significant for *interpersonal treatment* (*b* = −0.009, *p* = 0.033). Thus, only job applicants who interacted with an AI perceived lower *interpersonal treatment* (*b* = −0.009, *p* = 0.033) with increasing age.

Similarly, for the pooled models, we also find a negative effect of gender on the four dependent variables *outcome fairness* (*b* = −0.086, *p* = 0.047), *process fairness* (*b* = −0.228, *p* = 0.001), *interpersonal treatment* (*b* = −0.192, *p* = 0.002), and *recommendation intention* (*b* = −0.222, *p* = 0.002). Female job applicants have lower *fairness perceptions* and *recommendation intention* than male job applicants. This negative effect is consistent when looking at all scenarios, regardless of whether the decision was made by a human recruiter or an AI, but it is more pronounced in scenarios with an AI.

Except for vocational secondary school, job applicants’ higher educational attainment in the form of apprenticeship, high school, and a university degree have a negative effect on their perception of *outcome fairness*, *process fairness*, *interpersonal treatment*, and *recommendation intention*, with the effect being most salient for job applicants with a university degree. When job applicants have a university degree their perceived *outcome fairness* decrease, compared to the base scenario AI without explanation, by *b* = −0.179 (*p* = 0.012), *process fairness* by *b* = −0.343 (*p* = 0.002), *interpersonal treatment* by *b* = −0.369 (*p* = 0.001), *recommendation intention* by *b* = −0.399 (*p* = 0.001). These effects remain similar when only looking at job applicants who interacted with a human recruiter. However, for job applicants who interacted with an AI, effects of educational attainment are less consistent; while effects are present for high school on *outcome fairness* (see Model 1a), and for high school, and university education levels in case of perceived *process fairness* (see Model 2a), all other education effects disappear. Unlike other control variables, we find a positive effect of job applicants’ perceived discrimination on their *fairness perceptions* and *recommendation intention*. The more job applicants previously perceived discrimination in various (everyday) situations, the higher they perceived *outcome fairness* (*b* = 0.030, *p* = 0.033), *process fairness* (*b* = 0.037, *p* = 0.080), *interpersonal treatment* (*b* = 0.048, *p* = 0.017), and *recommendation intention* (*b* = 0.090, *p* < 0.001). When the decision was made by an AI, the negative effect remains only for *outcome fairness* (*b* = 0.049, *p* = 0.027) and *recommendation intention* (*b* = 0.075, *p* = 0.038). When the decision was made by a human recruiter, it remains only for *interpersonal treatment* (*b* = 0.072, *p* = 0.007) and *recommendation intention* (*b* = 0.105, *p* = 0.001).

## 6 Discussion, limitations and future research

Job applicants tend to react with aversion to the use of AI in personnel selection, often stemming from a low fairness perception of AI. Thus, this paper tackles one of the core challenges of adopting AI: How to deal with algorithm aversion in AI-based personnel selection so that job applicants perceive the personnel selection process as fair? Our study revealed that explanations have a positive impact on job applicants’ fairness perceptions for both AI and a human recruiter decision. Fairness perceptions and recommendation intention are highest for human recruiters providing an explanation for the job rejection and lowest for AI without explanations. Explanations can increase the positive evaluation of outcome fairness of AI decisions to the level of decisions made by human recruiters without explanation, and even surpass them regarding the evaluation of interpersonal treatment and recommendation intention.

### 6.1 Theoretical contribution

First, our study contributes to the research on algorithm aversion by specifically investigating the context of AI-based personnel selection. Our study shows for this case that explanations have a positive impact on fairness perceptions, whose strength, however, differs between the various components of the fairness construct as well as between AI and human recruiters. Previous research has shown that job applicants tend to prefer human recruiters over AI-based personnel selection and that explanations can help to close this gap (Tian et al., 2023). Our study provides a more nuanced picture by adopting the established theoretical framework of Gilliland et al. (2001) in the context of AI. Although organizational justice literature indicates that the fairness construct is composed of several components, such as outcome fairness or process fairness, previous studies concentrated on single outcome variables in the traditional personnel selection process (Jiang et al., 2022) or investigated them mainly from an AI perspective (Tian et al., 2023). By investigating and comparing the influence of explanation in both human-supported and AI-based personnel selection processes, our research findings build on and bridge studies focusing on the influence of perceived fairness in the context of either traditional or AI-based personnel selection processes. To the best of our knowledge, this is one of the first studies that has separately examined the influence of the absence and presence of explanations on the various components of fairness perceptions in the context of AI-based personnel selection. Thus, our findings offer a more nuanced picture than the typical ordinal ranking of previous studies and indicate that AI-provided explanations can counteract the aversion of AI in personnel selection. AI is often associated with low fairness, making algorithm aversion salient in various work contexts. Literature on algorithm aversion suggest specific framing factors (e.g., task factors) of the respective decision-making contexts to overcome algorithm aversion (Mahmud et al., 2022). By examining explanations’ effects on fairness perceptions in the specific example of personnel selection, our study addresses this requirement and offers domain-specific insights. Thus, it seems possible to specifically counteract the aversion to AI and increase the fairness perception and finally the intention to use AI in personnel selection (Fleiß et al., 2024). Furthermore, the effect varies between the distinct components of the fairness construct and between AI and human recruiters. When an explanation was provided, job applicants rated decisions made by a human highest on all fairness dimensions and AI without explanation lowest. Explanations can raise the positive outcome fairness ratings of AI-based decisions to the level of evaluations for decisions made by a human without explanation. Regarding interpersonal treatment and recommendation intention, the ratings of decisions made by AI surpass those of decisions made by human recruiters without explanation. The relatively positive evaluation of AI-based decisions with explanation in terms of interpersonal treatment is surprising, as this finding contradicts previous research in the field of computer science (Schlicker et al., 2021) that problematized users’ perceptions of poor interpersonal treatment provided by AI. Thus, our findings highlight the importance of bringing theoretical perspectives from the algorithm aversion literature into studying the influence of explanations on job applicants’ fairness perceptions, and of examining multiple dimensions of fairness, separately. As the research in this area is still underexplored (Hilliard et al., 2022), we offer first insights into the perceptions and behavior of individual job applicant groups when interacting with AI in personnel selection by demonstrating that sociodemographic characteristics such as age, gender, education, and discrimination experience of job applicants can influence their fairness perceptions and recommendation intentions.

Second, our study contributes to the literature on justice perceptions by showcasing that the fairness scales of Gilliland et al. (2001), designed for a traditional personnel selection process, are also applicable in an AI context. AI is increasingly used in personnel selection, which is controversially discussed in the context of organizational justice theory (Kaibel et al., 2019; Langer et al., 2019). However, to benefit from the use of AI, it is essential to be aware of the pitfalls associated with job applicants’ negative fairness perceptions and the impact of mitigating measures such as explanations on them (Köchling and Wehner, 2020). As fairness is characterized by multidimensionality and the interplay of its constructs, a holistic understanding and differentiated view of fairness perception requires the measurement of the individual fairness constructs (Ochmann et al., 2024). Organizational justice theory offers metrics for these, but they were primarily designed for human-supported processes, raising questions about their applicability to AI-based processes (Ochmann et al., 2024). Previous research on AI has focused on investigating the issue of bias from a technical perspective, with little attention paid to job applicant’s fairness perception of AI-based personnel selection of job applicants and its measurement (Köchling and Wehner, 2020). Conducted research on fairness perceptions is generally limited to certain fairness constructs, with only a few such as Ochmann et al. (2024) indicating the applicability of all fairness constructs proposed by organizational justice theory to the fairness assessment of AI. We address this gap and build on those studies by replicating and extending Gilliland et al. (2001) and demonstrating the applicability of its fairness scales for the various fairness constructs in the context of AI-based personnel selection.

### 6.2 Practical implications

Our findings indicate that providing explanations is a promising measure to mitigate job applicants’ aversion to AI and thus build a positive and long-term trust relationship between (future) employees and the organization at the earliest possible stage. Previous research, such as Figueroa-Armijos et al. (2023), has shown that the way AI is perceived by job applicants during the selection process influences whether they consider the hiring organization to be trustworthy. Since perceived fairness is linked to trust in management and organization (Komodromos, 2014), a person’s trust level is built on fairness treatment. However, when AI is used in personnel selection, job applicants often perceive the process as less fair (Zhang and Yencha, 2022), leading to an aversion towards AI. Negative assumptions about AI reduce employees’ intentions to use it (Malin et al., 2023). As our findings demonstrate, AI-provided explanations can increase perceived fairness during the personnel selection process, which has a positive impact on trust in AI and the organization. The fairer AI is perceived, the more it is viewed as trustworthy, which leads to a higher willingness to accept AI-based work systems (Shulner-Tala et al., 2023) and affects how individuals use and rely on them (Knickrehm et al., 2023). Since fairness perceptions during the selection process affect the reapplication intentions and subsequent work performance of job applicants, it can be assumed that job applicants who have been rejected in the context of an AI-based personnel selection, with a higher perception of fairness generated by explanations, are more likely to reapply to the same organization and show a higher work performance in the job.

Our study hints at factors that need to be considered when designing AI systems so that they are perceived as fair. Low fairness perception and aversion towards AI are most evident in work contexts in which AI performs tasks that involve subjective evaluation (Castelo et al., 2019) or moral decision-making (Lee, 2018). By comparing personnel selection scenarios without and with AI-provided explanations, our findings highlight that XAI contributes to a positive fairness perception in such an algorithm-aversion-prone work setting. Hence, an explanatory component is an essential design feature of (X)AI systems that shapes fairness perceptions (Hyesun et al., 2023).

Based on our findings, it can be concluded that job applicants’ fairness perceptions, even if they have been rejected, increase with XAI. Leaving a positive impression of the personnel selection process is beneficial for organizations for several reasons. Apart from personnel selection, AI can take over various tasks in numerous other organizational contexts, and it cannot therefore be ruled out that job applicants who have been selected by organizations with the assistance of AI will themselves interact or work with AI systems in their later position at the future employer. Since even rejected job applicants who have had a positive experience during the selection process are more likely to have trust in the organization and are willing to reapply, it can be assumed that they will reapply for another job for which they are highly qualified for and are more willing to work with AI systems in their later job.

The issue of algorithm aversion relating to a low fairness perception is currently being discussed in the context of AI regulation. Various stakeholders are increasingly calling for AI certification in high-risk application areas, such as personnel selection, to ensure fairness in AI-based personnel selection (Hunkenschroer and Luetge, 2022). Our findings that the fairness scales of Gilliland et al. (2001), which were developed for a traditional personnel selection process, can also be applied in an AI context is relevant to the interdisciplinary debate on the certification of AI applications. Given that there is no AI certification framework to date and the fairness scales of Gilliland et al. (2001) measure various dimensions of fairness separately, we recommend using them as measurement tools.

This paper has two major limitations. First, we examined the influence of explanations justifying why the selected person was most qualified for the open job position, on job applicants’ fairness perceptions and recommendation intention, while Gilliland et al. (2001) tested the influence of two additional types of explanations. Our study focuses on this type of explanation since studies, such as Fleiß et al. (2024), indicate that such organizational factors are the most salient in the case of AI-provided explanations. However, examining the influence of other types of explanations on potential job applicants’ fairness perceptions in AI-based personnel selection is a promising direction for future research. Second, we conducted a scenario-based vignette-style method, limiting the extent to which job applicants’ responses were examined in an actual application context. Studies, such as Taylor (2006), state that scenarios are an adequate method to capture attitudes, perceptions, and behavior from real-life situations. To extend our findings, research on the influence of explanations on job applicants’ fairness perceptions in AI-based personnel selection in real application contexts is needed.

## 7 Conclusion

Organizations’ intentions to adopt AI are overshadowed by humans’ aversion to it. This issue is also pertinent in personnel selection, as job applicants raise concerns about the fairness of AI-based procedures. This study sheds light on how AI-based decision-making can be designed so that job applicants perceive the personnel selection process as fair. Our findings show that in AI-based personnel selection processes, explanations can enhance job applicants’ perceptions of outcome fairness, process fairness, interpersonal treatment, and recommendation intentions. We are confident that our findings will serve as a starting point for the design of job applicant-tailored AI systems and strategies for the successful adoption of AI in personnel selection by increasing fairness perception and mitigating algorithm aversion and thus reducing resistance to AI in the work environment.

## Data Availability

The original contributions presented in the study are included in the article/supplementary material, further inquiries can be directed to the corresponding author.

## References

[ref1] Acikgoz Y. Davison K. H. Compagnone M. Laske M. (2020). Justice perceptions of artificial intelligence in selection. Int. J. Sel. Assess. 28, 399–416. doi: 10.1111/ijsa.12306

[ref2] Berger B. Adam M. Rühr A. Benlian A. (2021). Watch me improve—algorithm aversion and demonstrating the ability to learn. Bus. Inf. Syst. Eng. 63, 55–68. doi: 10.1007/s12599-020-00678-5

[ref3] Burton J. W. Stein M.-K. Blegind Jensen T. (2020). A systematic review of algorithm aversion in augmented decision making. J. Behav. Decis. Making 33, 220–239. doi: 10.1002/bdm.2155

[ref4] Cai F. Zhang J. Zhang L. (2024). The impact of artificial intelligence replacing humans in making human resource management decisions on fairness: a case of resume screening. Sustainability 16:3840. doi: 10.3390/su16093840

[ref5] Castelo N. Bos M. W. Lehmann D. R. (2019). Task-dependent algorithm aversion. J. Mark. Res. 56, 809–825. doi: 10.1177/0022243719851788

[ref6] Castelvecchi D. (2016). Can we open the black box of AI? Nature 538, 20–23. doi: 10.1038/538020a 27708329

[ref7] Czernietzki C. Märtins J. Westmattelmann D. Grotenhermen J.-G. Oldeweme A. (2023). Navigating AI in personnel selection: a scenario-based study on applicants’ perceptions. International Conference on Information Systems

[ref8] Daschner S. Obermaier R. (2022). Algorithm aversion? On the influence of advice accuracy on trust in algorithmic advice. J. Decis. Syst. 31, 77–97. doi: 10.1080/12460125.2022.2070951

[ref9] Diederich S. Brendel A. B. Kolbe L. M. (2020). Designing anthropomorphic enterprise conversational agents. Bus. Inf. Syst. Eng. 62, 193–209. doi: 10.1007/s12599-020-00639-y

[ref10] Fernández-Martínez C. Fernández A. (2020). AI and recruiting software: ethical and legal implications. Paladyn 11, 199–216. doi: 10.1515/pjbr-2020-0030

[ref11] Figueroa-Armijos M. Clark B. B. da Motta Veiga S. P. (2023). Ethical perceptions of AI in hiring and organizational trust: the role of performance expectancy and social influence. J. Bus. Ethics 186, 179–197. doi: 10.1007/s10551-022-05166-2

[ref12] Fleiß J. Bäck E. Thalmann S. (2024). Mitigating algorithm aversion in recruiting: a study on explainable AI for conversational agents ACM SIGMIS Database: the DATABASE for Advances in Information Systems. 56–87

[ref13] Folger R. Cropanzano R. (1998). Organizational justice and human resource management. Thousand Oaks, CA: SAGE.

[ref14] Gashi M. Vuković M. Jekic N. Thalmann S. Holzinger A. Jean-Quartier C.. (2022). State-of-the-art explainability methods with focus on visual analytics showcased by glioma classification. BioMedInformatics 2, 139–158. doi: 10.3390/biomedinformatics2010009

[ref15] Gilliland S. W. (1993). The perceived fairness of selection systems: an organizational justice perspective. Acad. Manag. Rev. 18, 694–734. doi: 10.2307/258595

[ref16] Gilliland S. W. Groth M. Baker R. C. I. Dew A. F. Polly L. M. Langdon J. C. (2001). Improving applicants’ reactions to rejection letters: an application of fairness theory. Pers. Psychol. 54, 669–703. doi: 10.1111/j.1744-6570.2001.tb00227.x

[ref17] Haque A. B. Islam A. N. Mikalef P. (2023). Explainable artificial intelligence (XAI) from a user perspective: a synthesis of prior literature and problematizing avenues for future research. Technol. Forecast. Soc. Chang. 168:122120. doi: 10.1016/j.techfore.2022.122120

[ref18] Hilliard A. Guenole N. Leutner F. (2022). Robots are judging me: perceived fairness of algorithmic recruitment tools. Front. Psychol. 13:940456. doi: 10.3389/fpsyg.2022.940456, PMID: 35959005 PMC9358218

[ref19] Hunkenschroer A. L. Luetge C. (2022). Ethics of AI-enabled recruiting and selection: a review and research agenda. J. Bus. Ethics 178, 977–1007. doi: 10.1007/s10551-022-05049-6

[ref20] Hyesun C. Prabu D. Arun R. (2023). Trust in AI and its role in the acceptance of AI technologies. Int. J. Hum. Comput. Interact. 39, 1727–1739. doi: 10.1080/10447318.2022.2050543

[ref21] Jiang L. J. Qin X. Dong X. Chen C. Liao W. (2022). How and when AI-human order influences procedural justice in a multistage decision-making process. Academy of Management Annual Meeting Proceedings

[ref22] Jussupow E. Benbasat I. Heinzl A. (2020). Why are we averse towards algorithms? A comprehensive literature review on algorithm aversion. European Conference on Information Systems

[ref23] Kaibel C. Koch-Bayram I. Biemann T. Mühlenbock M. (2019). Applicant perceptions of hiring algorithms-uniqueness and discrimination experiences as moderators. Academy of Management Proceedings

[ref24] Kim D. Song Y. Kim S. Lee S. Wu Y. Shin J.. (2023). How should the results of artificial intelligence be explained to users?—research on consumer preferences in user-centered explainable artificial intelligence. Technol. Forecast. Soc. Change 188:122343. doi: 10.1016/j.techfore.2023.122343

[ref25] Kleiner R. L. Hartwich N. J. Antons D. (2023). Enhancing perceived fairness of AI-based personnel selection procedures: the role of AI certification. International Conference on Information Systems

[ref26] Knickrehm C. Voss M. Barton M.-C. (2023). Can you trust me? Reviewing more than three decades of AI trust literature. European Conference on Information Systems

[ref27] Koch-Bayram I. F. Kaibel C. (2023). Algorithms in personnel selection, applicants’ attributions about organizations’ intents and organizational attractiveness: an experimental study. Hum. Resour. Manag. J. 34, 733–752. doi: 10.1111/1748-8583.12528

[ref28] Köchling A. Wehner M. C. (2020). Discriminated by an algorithm: a systematic review of discrimination and fairness by algorithmic decision-making in the context of HR recruitment and HR development. Bus. Res. 13, 795–848. doi: 10.1007/s40685-020-00134-w

[ref29] Köchling A. Wehner M. C. Warkocz J. (2022). Can I show my skills? Affective responses to artificial intelligence in the recruitment process. Rev. Manag. Sci. 17, 2109–2138. doi: 10.1007/s11846-021-00514-4

[ref30] Komodromos M. (2014). Employees’ perceptions of trust, fairness, and the management of change in three private universities in Cyprus. J. Hum. Resour. Manag. Labor Stud. 2, 35–54. Available at: https://jhrmls.thebrpi.org/journals/jhrmls/Vol_2_No_2_June_2014/3.pdf

[ref31] Langer M. König C. J. Papathanasiou M. (2019). Highly automated job interviews: acceptance under the influence of stakes. Int. J. Sel. Assess. 27, 217–234. doi: 10.1111/ijsa.12246

[ref32] Laurim V. Arpaci S. Prommegger B. Krcmar H. (2021). Computer, whom should I hire?—acceptance criteria for artificial intelligence in the recruitment process. Hawaii International Conference on System Sciences

[ref33] Lavanchy M. Reichert P. Narayanan J. Savani K. (2023). Applicants’ fairness perceptions of algorithm-driven hiring procedures. J. Bus. Ethics 188, 125–150. doi: 10.1007/s10551-022-05320-w

[ref34] Lee M. K. (2018). Understanding perception of algorithmic decisions: fairness, trust, and emotion in response to algorithmic management. Big Data Soc. 5, 1–16. doi: 10.1177/2053951718756684

[ref35] Lee M. K. Baykal S. (2017). Algorithmic mediation in group decisions: fairness perceptions of algorithmically mediated vs. discussion-based social division. ACM Conference on Computer Supported Cooperative Work and Social Computing

[ref36] Mahmud H. Islam N. A. Ahmed S. I. Smolander K. (2022). What influences algorithmic decision-making? A systematic literature review on algorithm aversion. Technol. Forecast. Soc. Change 175:121390. doi: 10.1016/j.techfore.2021.121390

[ref37] Malin C. Kupfer C. Fleiß J. Kubicek B. Thalmann S. (2023). In the AI of the beholder—a qualitative study of HR professionals’ beliefs about AI-based chatbots and decision support in candidate pre-selection. Adm. Sci. 13:231. doi: 10.3390/admsci13110231

[ref38] McLarty B. D. Whitman D. S. (2016). A dispositional approach to applicant reactions: examining core self-evaluations, behavioral intentions, and fairness perceptions. J. Bus. Psychol. 31, 141–153. doi: 10.1007/s10869-015-9405-x

[ref39] Mirowska A. Mesnet L. (2022). Preferring the devil you know: potential applicant reactions to artificial intelligence evaluation of interviews. Hum. Resour. Manag. J. 32, 364–383. doi: 10.1111/1748-8583.12393

[ref40] Nabi S. (2023). Comparative analysis of AI vs. human based hiring process: a survey. International Conference on Computational Intelligence and Knowledge Economy

[ref41] Narayanan D. Nagpal M. Mahak J. Schweitzer S. Cremer D. (2023). Fairness perceptions of artificial intelligence: a review and path forward. Int. J. Hum. Comput. Interact. 40, 4–23. doi: 10.1080/10447318.2023.2210890

[ref42] Newman D. T. Fasta N. J. Harmon D. J. (2020). When eliminating bias isn’t fair: algorithmic reductionism and procedural justice in human resource decisions. Organ. Behav. Hum. Decis. Process. 160, 149–167. doi: 10.1016/j.obhdp.2020.03.008

[ref43] Noble S. M. Foster L. L. Craig S. B. (2022). The procedural and interpersonal justice of automated application and resume screening. Int. J. Sel. Assess. 29, 139–153. doi: 10.1111/ijsa.12320

[ref44] Nørskov S. Damholdt M. F. Ulhøi J. P. Jensen M. B. Ess C. Seibt J. (2020). Applicant fairness perceptions of a robot-mediated job interview: a video vignette-based experimental survey. Front. Robot. AI 7:586263. doi: 10.3389/frobt.2020.586263, PMID: 33501344 PMC7805899

[ref45] Ochmann J. Michels L. Tiefenbeck V. Maier C. Lammer S. (2024). Perceived algorithmic fairness: an empirical study of transparency and anthropomorphism in algorithmic recruiting. Inf. Syst. J. 34, 384–414. doi: 10.1111/isj.12482

[ref46] Öttning S. K. Maier G. W. (2018). The importance of procedural justice in human–machine interactions: intelligent systems as new decision agents in organizations. Comput. Human Behav. 89, 27–39. doi: 10.1016/j.chb.2018.07.022

[ref47] Rigotti C. Fosch-Villaronga E. (2024). Fairness, AI & recruitment. Comput. Law Secur. Rev. 53:105966. doi: 10.1016/j.clsr.2024.105966

[ref48] Schlicker N. Langer M. Öttning S. K. Baum K. B. König C. J. Wallach D. (2021). What to expect from opening up ‘black boxes’? Comparing perceptions of justice between human and automated agents. Comput. Human Behav. 122:106837. doi: 10.1016/j.chb.2021.106837

[ref49] Searle R. Skinner D. (2011). Trust and human resource management. Cheltenham: Edward Elgar.

[ref50] Shulner-Tala A. Kuflika T. Klinger D. (2023). Enhancing fairness perception–towards human-centred AI and personalized explanations understanding the factors influencing laypeople’s fairness perceptions of algorithmic decisions. Int. J. Hum. Comput. Interact. 39, 1455–1482. doi: 10.1080/10447318.2022.2095705

[ref51] Suen H.-Y. Chen M. Y.-C. Lu M. Y.-C. (2019). Does the use of synchrony and artificial intelligence in video interviews affect interview ratings and applicant attitudes? Comput. Human Behav. 98, 93–101. doi: 10.1016/j.chb.2019.04.012

[ref52] Taylor B. J. (2006). Factorial surveys: using vignettes to study professional judgement. Br. J. Soc. Work 36, 1187–1207. doi: 10.1093/bjsw/bch345

[ref53] Tian Y. Cloudy M. Xu D. J. Shaoyi S. (2023). Exploring the role of AI explanations in delivering rejection messages: a comparative analysis of organizational justice perceptions between HR and AI. International Conference on Information Systems

[ref54] van Esch P. Black J. S. (2019). Factors that influence new generation candidates to engage with and complete digital, AI-enabled recruiting. Bus. Horiz. 62, 729–739. doi: 10.1016/j.bushor.2019.07.004

[ref55] Younis R. A. A. Salama M. R. Rashwan M. M. S. (2024). How does perception of artificial intelligence-user interaction (PAIUI) impact organizational attractiveness among external users? An empirical study testing the mediating variables. Comput. Hum. Behav. Artif. Hum. 2:100048. doi: 10.1016/j.chbah.2024.100048

[ref56] Zhang L. Yencha C. (2022). Examining perceptions towards hiring algorithms. Technol. Soc. 68:101848. doi: 10.1016/j.techsoc.2021.101848

